# Supplementary material for: C1q drives neural stem cell quiescence by regulating cell cycle and metabolism through BAI1
Source: Nat Commun. 2025 Dec 11;16:11386. doi: 10.1038/s41467-025-66185-x (PMC12738564; doi:10.1038/s41467-025-66185-x)
Supplement: Supplementary file 1 — Supplementary information [file 41467_2025_66185_MOESM1_ESM.pdf]

Piltti, KM et al. C1q drives neural stem cell quiescence by regulating cell cycle and metabolism through BAI1 - Supplementary information

Supplementary Information items:

Supplementary Methods

Supplementary Figure 1

Supplementary Figure 2

Supplementary Figure 3

Supplementary Figure 4

Supplementary Figure 5

Supplementary Figure 6

Supplementary Figure 7

Supplementary Table 1

Supplementary Table 2

SUPPLEMENTARY METHODS

Key Resources Table

REAGENT or RESOURCE	SOURCE	IDENTIFIER
**Antibodies**		
Cleaved Caspase-3(Asp175)(D3E9) mAb	Cell Signaling	9579<br>RRID:AB_10897512
Anti-C1q nAb (goat anti-human)	Quidel	A031
Anti-C1q antibody	Abcam	Ab71940<br>RRID AB_10711046
Complement Component C1s Antibody (M241)	Novus Biologicals	NBP1-52122<br>RRID:AB_11043800
Rabbit Polyclonal BAI1 antibody	Abcam	Ab135907
Human BAI1 neutralizing antibody	R&D Systems	AF4969<br>RRID:AB_10718844
BAI1 polyclonal antibody	LSBio	LS-C120632<br>RRID:AB_10801493
BAI1 antibody	Novus Biologicals	NB110-81586<br>RRID:AB_1144873
BAI1 polyclonal antibody	Thermo Fisher Scientific	PA5-102069<br>RRID:AB_2815896
MDM2 (D1V2Z) rabbit mAb	Cell Signaling Technology	86934<br>RRID:AB_2784534
MDM2 Antibody (SMP14)	Santa Cruz Biotechnology	Sc-965<br>RRID:AB_627920
Recombinant Anti-Met (c-Met) antibody - N-terminal (ab51067)	Abcam	Ab51067<br>RRID AB_880695
Phospho-p53 (Ser15) (16G8) mouse mAb	Cell Signaling Technology	9286<br>RRID AB_331741
p53 antibody	Cell Signaling Technology	9282<br>RRID:AB_331476
Recombinant Anti-GC1qR (p32) antibody	Abcam	Ab24733<br>RRID:AB_448269
Tom20 (D8T4N) Rabbit mAb	Cell Signaling Technology	42406<br>RRID:AB_2687663

Anti-C3aR antibody	Abcam	Cat# ab103629<br>RRID:AB_10888127
Anti-human complement c3a (H13)	Millipore	CBL191<br>RRID:AB_93307
Anti-EGFR antibody (EP38y) rabbit mAb	Abcam	Cat# ab52894<br>RRID:AB_869579
MTCO1 monoclonal antibody (1D6E1A8) (anti-COX IV antibody	Thermo Fisher Scientific	459600<br>RRID:AB_10374492
Purified anti-Tubulin β3 (TUBB3)	Biolegend	801202<br>RRID:AB_2313773
Anti-Glial Fibrillary Acidic Protein antibody	Dako	Z 0334<br>AB_10013382<br>RRID:AB_10013382
Anti-Glial Fibrillary Acidic Protein δ antibody	Millipore	AB9598<br>RRID:AB_2315616
Recombinant anti-S100β antibody	Abcam	Ab52642<br>RRID:AB_882426
Anti-Vimentin antibody	Abcam	Ab24525<br>RRID:AB_778824
Anti-Nestin antibody	Abcam	Ab11306<br>RRID:AB_1640723
Human/mouse/rat SOX2 antibody	R&D Systems	AF2018<br>RRID:AB_355110
CD133/1 (AC133) PE, human antibody	Miltenyi Biotec	130-113-108<br>RRID:AB_244342
CD34 (AC136) FITC, human antibody	Miltenyi Biotec	130-113-178<br>RRID:AB_244350
Anti-β-Actin antibody, Mouse monoclonal	Sigma Aldrich	A1978<br>RRID:AB_476692
Anti-α tubulin antibody (DM1A)	Abcam	Ab7291<br>RRID:AB_2241126
Anti-STEM121	Takara	Y40410<br>RRID:AB_2801314
Anti-BrdU antibody	Abcam	Ab1893<br>RRID:AB_302659
Normal sheep IgG control	R&D Systems	5-001-A<br>RRID:AB_10141430

Normal goat IgG control	R&D Systems	AB-108-C RRID:AB_354267
Mouse IgG Isotype control	Thermo Fisher Scientific	Cat# 31903 RRID:AB_10959891
**Chemicals, peptides, and recombinant proteins**		
Complement Component C1q Human	MyBioSource	MBS147305
C1 Human	CompTech	A098
C1 inhibitor	Millipore	GF178
C3a anaphylatoxin	Complement Technology	A118
BAI1 overexpression lysate	Novus Biologicals	NBP2-08183
BAI1 empty vector negative control	Novus Biologicals	NBP2-08183
pHrodo™ Red Epidermal Growth Factor (EGF) Conjugate	Thermo Fisher Scientific	P35374
CellLight™ Early Endosomes-RFP, BacMam 2.0	Thermo Fisher Scientific	C10587
Human GC1qR / C1QBP Protein (Recombinant)	LSBio	LS-G3375-20
Recombinant Human BAI1 Protein, CF (His Tag)	Biotechne	4969-BA
Recombinant Human HABP1/C1qBP/gC1qR Protein (His Tag)	SinoBiological	11874-H08E
Hoechst 33342	Invitrogen	H3570
DAPI (4',6-Diamidino-2-Phenylindole, Dilactate)	Biolegend	422801
WGA, Alexa FluorTM 488 conjugate	Invitrogen	W11261
Hematoxylin	Sigma-Aldrich	MHS32
Mitotracker Red CMXRos	Thermo Fisher Scientific	M7512
Mitotracker CM-H2TMRos	Thermo Fisher Scientific	M7511
Carboxy-H2DCFDA	Thermo Fisher Scientific	C400
**Critical commercial assays/tools**		
PathScan intracellular signaling array kit	Cell Signaling	7744
RealTime-Glo Annexin V Multiplex Assay	Promega	JA1001

HQ Silver Enhancement Kit	Nanoprobes	2012
Duolink® In Situ PLA® Probe Anti-Rabbit PLUS	Millipore Sigma	duo92002
Duolink® In Situ PLA® Probe Anti-Mouse MINUS	Millipore Sigma	duo92004
BrdU Amersham Cell proliferation kit	GE healthcare	45-000-837
EdU Click-iT® EdU Imaging Kits	Invitrogen	C10038
Ez-link NHS-SS-Biotin	ThermoScientific	PI21441
EZ-Link™Sulfo-NHS-SS-Biotin	ThermoScientific	21331
Seahorse XF Cell Mito Stress Test Kit	Agilent Technologies	103015-100
QCM Chemotaxis Cell Migration Assay, 96-well	Millipore/ Chemicon	ECM510
Dynabeads™ His-Tag Isolation and Pulldown	Invitrogen	10103D
**Experimental models: Cell lines**		
Human neural stem cells	UC Irvine, Anderson AJ	BAI1 WT (UCI161) hNSC
Human neural stem cells	UC Irvine, Anderson AJ	BAI1 KO (UCI161) hNSC
Multipotent human CNS-derived stem cells	Uchida et al., 2000	Stem cells Inc 2491.2
Mouse neural stem cells	UC Irvine, Anderson AJ	Fucci-mNSC
**Experimental models: Organisms/strains**		
Rag1 mice	JAXmice	002216 - B6.129S7-Rag1 < tm1Mom>/J
**Recombinant DNA**		
Chimeric gRNA guide	https://www.appliedstemcell.com/	Custom made
Chimeric gRNA +hspCAS9 co-expression vector	https://www.appliedstemcell.com/	Custom made
**Software and algorithms**		
Imaris version 9.1.2	Andor Technology Ltd	RRID: SCR_007370

Fiji / ImageJ	ImageJ	RRID: SCR_003070
StereoInvestigator	Microbrightfield	
SnapGene	SnapGene	RRID:SCR_015052
Amnis Inspire	Luminex	
Amnis Ideas version 6.2	Luminex	
MaxQuant version 1.6.0.16	MaxQuant	RRID:SCR_014485
DEP version 1.28	Bioconductor	RRID:SCR_023090
FastQC	Babraham Institute	RRID:SCR_014583
SortMeRNA	LIFL	RRID:SCR_014402
Trimmomatic	Max Planck Institute	RRID:SCR_011848
EdgeR	Bioconductor	RRID:SCR_012802
Gene Set Enrichment Analysis (GSEA)	Broad Institute	Cambridge RRID:SCR_003199
Molecular Signatures Database (MSigDB)	Broad Institute	Cambridge RRID:SCR_016863
SimFCS software	Laboratory for Fluorescence Dy namics	UC Irvine
CellProfiler version 4.2.1	CellProfiler	RRID:SCR_007358
MitoMo Integrated Mitochondrial image analysis software	Visualization and Intelligent Systems Laboratory	UC Riverside
Matlab	MathWorks	RRID:SCR_001622
Agilent Seahorse Wave version 2.6.1	Agilent Technologies	RRID:SCR_024491
Prism version 10	GraphPad	RRID:SCR_002798

Neurosphere dissociation for proliferation, self-renewal, and RealTime-Glo Annexin V multiplex assays

Human and mouse NSC were cultured as free-floating neurospheres in X-vivo (Lonza, 04-744Q) or Stemline (Sigma-Aldrich, S3194-500) growth medium (GM) as previously described [95]. Neurospheres were dissociated into single cells by mixing the cell clusters in Mg and Ca-free Dulbecco's Phosphate Buffered Solution (DPBS$^{-/-}$; Invitrogen, 14190-250) supplemented with 10.5µg/mL of Liberase^{TM} Research Grade (Roche, 50-100-3280) and 0.1% Human serum

albumin (HSA 25%; LGC Clinical Diagnostic Inc., 1880-0004) at 37 ℃ 1400 rpm for 6min using Thermomixer (Eppendorf) followed by mechanical dissociation by pipetting. After DPBS$^{-/-}$/ 0.1% HSA wash, any remaining cell clusters were removed from the single-cell solution using a 40μm cell strainer (BD Falcon). For the assays requiring monolayer culture, the single cells were plated on PLO (5 μg/mL; Sigma-Aldrich, P3655) and laminin (10μg/mL; Invitrogen, 23017-015) coated 8-well chamber slides at a density of 15,000 cells/well in X-vivo GM overnight. The following day, the cells were changed into X-vivo DM without (non-treated control) or with C1q. In all experiments, filtered C1q stock was directly added into medium on cell culture vessels and gently mixed to achieve final sample concentrations ranging from 0.1nM to 300nM.

RNA extraction, quantity and quality

RNA was extracted using RNeasy Kit (Qiagen, 74104) with Qiashreder (Qiagen, 79654) column step for cell homogenization, followed by DNase treatment and purification using a DNAse Free kit (Ambion, AM1906) to ensure high-quality RNA samples. The quantity and quality of the purified RNA was performed using a Qubit 2.0 Fluorometer (Invitrogen) and Agilent Technologies Bioanalyzer 2100.

CRISPR Cas9 hNSC BAI1 genetic deletion and BAI1 KO hNSC characterization

We developed an alternative two-stage genetic modification strategy to derive BAI1 WT and KO hNSC using fluorescence activated cell sorting (FACS) after homology directed repair (HDR) mediated by CRISPR cas9. This genetic modification strategy entailed two rounds of genome editing that both used FACS to isolate edited cells based on the presence or absence of color. In the first round of editing, using a custom BAI1 donor template (Supplementary Fig.2a) and a BAI1 CRISPR plasmid (Applied Stem Cells, cat no. P0049-P001), we introduced premature stop codons into first open reading frame of BAI1 within exon 2 - along with a FLOXED CMV-mScarlet-P2A-PuroR cassette; the introduction of this genetic modification causes edited cells to be mScarlet-positive and BAI1 protein is truncated at amino acid 52 of 1584 amino acids total, reducing the size of BAI1 by 96.7% to effectively create precise genetic KO in hNSC (Supplementary Fig.2b). In the second round of editing, CRE recombinase is added to the mScarlet-postive BAI1 KO hNSC to remove the selection cassette (Supplementary Fig.2b), causing the BAI1 KO hNSC to become mScarlet-negative.

The cas9-expressing-BAI1 Guide RNA plasmid (gRNA sequence in Supplementary Fig.2c) was custom-generated and validated in human HEK293 cells by Applied Stem Cells (cat no. P0049-P001). The BAI1 donor plasmid (Supplementary Fig.2a) was constructed in-house from 4 PCR-amplified segments using NEB assembly (cat no. E2621S) and custom IDT primers according to the manufacturer instructions. Briefly, the 4 PCR-amplified segments were: the left and right arms of homology (AOH) needed to integrate the genetic modifications into the BAI1 genomic locus, the 'Stop-Floxed-Selection Cassette 'which introduced 3 premature stop codons into the start of the BAI1 open reading frame and a mScarlet-Puromycin selection cassette flanked by LOXP sites, and the last segment was the plasmid backbone. The left and right AOH were amplified from a BAI1 topo plasmid that was generated in-house by PCR-amplifying a 1441 base pair segment of the BAI1 genomic locus from the genomic DNA of unmodified human neural stem

cells using a topo cloning kit (cat no. K2875J10) according to the manufacturer instructions. The last 2 segments were amplified from a pCMV-mScarlet-P2A-Puromycin plasmid (Addgene). The final assembled BAI1 donor plasmid was verified through sanger sequencing and double restriction enzyme digestion with XhoI and XmnI - which produced 3 fragments for successfully assembled clones: 1153 bp, 1293bp, and 4438bp. Specifically, in the first stage of our genetic-modification strategy, BAI1 KO hNSC lines were generated by co-transfecting BAI1 donor and gRNA plasmid using nucleofection into UCI161 hNSC line at 65% efficiency (Lonza, Nucleofector buffer L). Two weeks post-transfection, mScarlet- (i.e. BAI1 WT) and mScarlet$^+$ (i.e. BAI1 KO) hNSC were separated and enriched by FACS. The gating parameters were highly stringent to avoid any heterozygous cell populations (low expression of BAI1) within the sorted cell pools.

In the second stage, 1-2 months post-transfection, the BAI1 KO cells were enriched for mScarlet$^+$ for a second time before they were transiently transfected with a transient expression vector carrying CRE-GFP to silence mScarlet. 2 weeks post-CRE, homozygous insertion of the frame-shift premature stop codons in BAI1 KO hNSC was verified via PCR of genomic DNA and Sanger sequencing (Supplementary Fig.2d,e). Stable loss of total BAI1 protein (174 kDa) expression was confirmed by Western blotting (Fig.3b), and functional-level, by performing PLA specific for the protein-protein interaction of BAI1 with its ligand, C1q, in both BAI1 KO and WT cells (Fig.3c-d). Two different BAI1 KO lines were generated with the same method. No substantial differences between the cell lines generated were detected regarding their CD133+ content, multipotency, migration, or proliferative capacity. Follow-up experiments *in vitro* were done at least independent experimental triplicates. BAI1 WT and BAI1 KO hNSC exhibited sustained normal karyotype (Cell Line Genetics), high CD133+ stem cell proportions, stable growth rate under *in vitro* growth conditions, migration response, and multipotency in neural lineage differentiation (Supplementary Fig.2f-n) demonstrating that both cell lines retained normal hNSC characteristics. Flow cytometric analysis of CD133+ cell proportion, chemotaxis transwell assays, and *in vitro* differentiation tests were conducted as previously described[20]. The doubling time, defined as the number of hours required for a cell population to double under standard in vitro growth conditions, was calculated as:

$$\text{Doubling time} = \frac{t \times \log(2)}{\log(N_f) - \log(N_i)}$$

where t is the culture duration (hours), $N_{(f)}$ is the final cell number, and $N_{(i)}$ is the initial cell number. Doubling times per each group per each biologically independent experiment and timepoint were tested using ROUT (Q = 10%) outlier test, Prism version 10 (GraphPad). For chemotaxis transwell assays and *in vitro* differentiation analysis, all biologically independent experiments were performed at least with technical duplicates. Transwell assay wells with either excessive or inadequate amounts of detachment buffer due to technical errors during the test were excluded from the analysis. Exclusions are listed in the Source data. For CD133 flow cytometry and population doubling time, each biologically independent experiment consists of cells collected from one flask. All analyses were performed by investigators blinded to experimental conditions/groups.

Human BAI1 antibody (R&D systems, AF4969) used for analyzing total BAI1 protein in BAI1 WT vs. KO hNSC in non-reducing Western blots (Fig. 3b) recognizes the correct BAI1 band (174 kDa) in BAI1 overexpression HEK293T cell lysate (+) (Novus Biologicals, NBP2-08183) (Supplementary Fig. 3a). In BAI1 overexpressing HEK293T cells, full-length BAI1 is present at two different molecular weights due to post translational modifications. Manufacturer of the antibody reports <2% cross-reactivity with recombinant human BAI3 at 170-130 kDa molecular weight.

Proximity ligation assays and signal quantification

hNSC seeding density for PLA-assays was 20,000 cells/well in GM. After treatment, cells were fixed with PFA 4% for 5min, washed with Hank's Buffer Salt Solution with Ca and Mg (HBSS$^{+/+}$; Thermo Fisher Scientific) and stained with WGA-conjugate 488 (Invitrogen) for 10min in RT. Next, the cells were washed, and permeabilized with 0.01% Triton X (Sigma Aldrich) and 2% Donkey Serum (Jackson Immunoresearch) in HBSS$^{+/+}$ for 4min. PLA was performed using Duolink® In Situ PLA® Probe Anti-Mouse MINUS (Sigma-Aldrich, DUO92004,), Duolink® In Situ PLA® Probe Anti-Rabbit PLUS (Sigma-Aldrich, DUO92002), and Duolink® In Situ Detection Reagents Red (Sigma-Aldrich, DUO92008), following the manufacturer instructions. 8 random pictures per condition were captured using BZ-X All-in-One Fluorescence Microscope (Keyence) or LSM 900 (Zeiss) as z-stacks of optical slices in 0.3 µm intervals using 60x objective. Red PLA punctae were quantified using Imaris version 9.1.2, and the data is represented as total punctae per cells with positive signal. PLA was pseudo-colored as magneta and WGA white in the example pictures.

Ligand/receptor internalization, colocalization, and spot count analysis using imaging flow cytometry

For matched ligand-G protein-coupled receptor C3a-C3aR complex internalization analysis, hNSC were treated with purified human C3a anaphylatoxin (Complement Technology, A118) at 100nM concentration or an equal volume of GM (non-treated control) for 5min, 15min, or 30min. For matched ligand-tyrosine kinase receptor EGF-EGFR complex internalization analysis, hNSC were treated with pHrodo™ red Epidermal Growth Factor (EGF) Conjugate (Thermo Fisher Scientific, P35374) at 40ng/mL concentration or an equal volume of GM (non-treated control) for 3min. C1q-C3aR a mis-matched ligand-receptor staining was used as a negative control.

For ligand/receptor internalization analysis, cells were eroded using a component mask (Fig.4e) of 7-pixel (Fig.4b,c,o-q, and Supplementary Fig.4b-d,f-h) or 3-pixel on a brightfield channel (Figs.4d,u and 5a,b). Cells with a ligand/receptor internalization score ≥0.5 were gated as internalized. For colocalization analysis, cell population exhibiting ligand and receptor internalization were plotted on a histogram using bright detail similarity R3 feature designed to specifically to compare the small bright image detail of two images. This feature is the log transformed Pearson's correlation coefficient of the localized bright spots with a radius of 3 pixels or less within the masked area in the two input images. Since the bright spots in the two images are either correlated (in the same spatial location) or uncorrelated (in different spatial locations),

the correlation coefficient varies between 0 (uncorrelated) and 1 (perfect correlation) and does not assume negative values. The coefficient is log transformed to increase the dynamic range between (0, infinity). In this study, mean bright detail similarity R3 score (co-localization score) ≥1.5 was considered colocalized (Fig.4d,o,u, and Supplementary Figs.3c and 4d,h). Spot count feature (Supplementary Figs.3c,4n) was used for measuring the quantity of internalized protein per cell (Figs.4q,5b).

LC-MS/MS and data analysis

Peptide digests were analyzed using an UltiMate 3000 RSLC system coupled in-line to an Orbitrap Fusion Lumos mass spectrometer (Thermo Scientific). Reverse-phase separation was performed on a 50 cm x 75 µm I.D. Acclaim® PepMap RSLC column. Peptides were eluted using a gradient of 4% to 22% B over 70min at a flow rate of 300nL/min (solvent A: 100% H2O, 0.1% formic acid; solvent B: 100% acetonitrile, 0.1% formic acid). Each cycle consisted of one full Fourier transform scan mass spectrum (375–1500 m/z, resolution of 120,000 at m/z 400) followed by data-dependent MS/MS scans acquired in the linear ion with HCD at NCE 30% trap at top speed for 3s. Target ions already selected for MS/MS were dynamically excluded for 30s. Protein identification and label-free quantitation were carried out using MaxQuant as described[109]. Raw spectrometric files were searched using MaxQuant version 1.6.0.16 against a FASTA of the complete human proteome obtained from SwissProt Feb 2020 version. The first search peptide tolerance was set to 20 ppm, with main search peptide tolerance set to 4.5 ppm. Trypsin was set as the digestive enzyme with max 2 missed cleavages. Methione oxidation and protein N-terminal acetylation were wet as variable modifications, while cysteine carbamidomethylation was set as a fixed modification. Peptide spectra match and protein FDRs were both set as 0.01. The generated raw mass spectrometry data was preprocessed and analyzed by Differential Enrichment Pipeline package (DEP) version 1.22 (Bioconductor)[110]. For quality control included filtering proteins with high missing values, assessing reproducibility, and addressing batch effect, the tool's default settings were applied. Quantitative values were normalized using variance stabilizing transformation (VST) to minimize technical variation and improve comparability across samples. For differential expression analysis, the samples treated with biotinylated C1q per each biologically independent experiment were pooled together and protein-wise linear models combined with empirical Bayesian statistics, as implemented in the DEP package, were used to identify differentially expressed proteins between two conditions bait versus non-treated control. Adjusted p-values were calculated using the Benjamini-Hochberg method, with proteins showing an FDR-adjusted p-value ≤0.05 considered significantly differentially expressed. The significant proteins are listed in the Source data. Hypergeometric tests in SubCellViz R package[111] was employed to analyze the subcellular localization of the differentially expressed proteins to illustrate protein distribution and identify compartment-specific functional patterns. Protein identifiers were annotated to subcellular compartments using Gene Ontology Cellular Component (GO-CC) and UniProt databases. Enrichment analysis was performed using hypergeometric tests, and compartments with FDR-adjusted p-values ≤ 0.05 were identified as significantly enriched. All analyses were performed by investigators blinded to experimental conditions/groups.

FLIM phasor data

FLIM data was acquired and processed by the SimFCS software (Laboratory for Fluorescence Dynamics) as previously described[65, 66, 67]. FLIM calibration of the system was performed by measuring the known lifetime of fluorescein with a single exponential of 4.04 ns. Phasor average values of each cell per experimental timepoint were calculated and represented as scatter plots. Every FLIM image was acquired over 10 frames of the same field of view. Every pixel of the FLIM image was transformed in one pixel into the phasor plot, and coordinates for g and s in the phasor plot were calculated from the fluorescence intensity decay of each pixel of the image. G coordinate reflects the NADH state.

Seahorse XF24 extracellular flux analysis

hNSC metabolic status after the treatment was determined using Seahorse XF24 Cell Mito Stress Test Kit (Agilent Technologies) was used to determine hNSC metabolic status according to the manufacturer instructions. Briefly, cartridge was filled with XF calibrant 1mL/well to get hydrated at 37°C in a non-CO^2 incubator overnight. Cells were carefully washed with XF medium supplemented with 5mM sodium pyruvate, 2.5mM L-glutamine and 17.5mM glucose and incubated in XF medium at 37°C in a non-CO^2 incubator for 1h. Meanwhile, sensor cartridge was prepared and Oligomycin, FCCP, and Rotenone/Antimycin (Agilent Technologies) were added to their corresponding ports for final concentrations of 10µM. After calibration, the plate with non-treated and treated hNSC and blank wells was inserted into Seahorse XF24 extracellular flux analyzer (Agilent Technologies). Analysis program included a step of equilibration followed by basal, post-oligomycin injection, post-FCCP injection, and post-Rotenone/Antimycin measurements. Each of these 4 steps comprises three loops of 3min mixing, 2min waiting, and 3min measuring. Basal OCR and ECAR are mean of three basal reads per group per each independent experiment.

Mitochondrial membrane potential and reactive oxygen species

Time-course analyses of mitochondrial membrane potential was performed using reduced MitoTracker® Orange CM-H2TMRos dye (Invitrogen, Cat. no. M7511) according to the manufacturer instructions. Briefly, hNSC monolayers were exposed to C1q either at 0.1nM, 1nM, or 200nM concentration vs. non-treated control or temperature-inactivated C1q control either for 30min, 2h, 24h or 48h. Next, the cells were detached into single cells, centrifuged, resuspended into Eppendorf tubes in prewarmed GM with 100nM MitoTracker® Orange CM-H2TMRos and incubated for 30min under growth conditions at +37°C. The cells were centrifuged and resuspended into warm GM with 4% PFA. After fixation, median fluorescence of CM-H2TMRos DCFDA intensities were analyzed using a BD FACSDiva 9.0 Flow Cytometer (BD Biosciences). Gating for the flow cytometry analyses were set using unstained negative control cells and single labeled cells. Time-course data was collected from one to four biologically independent experiments with one sample well per each. Normalized CM-H2TMRos in each group per each independent experimental replicate were tested for outliers using Grubb's outlier test (Alpha 0.2) Prism version 10. Outliers are listed in the Source data.

Time-course analyses of ROS production were studied using negatively charged ROS-indicator carboxy-H2DCFDA (Invitrogen, Cat. no. C400) according to the manufacturer instructions. Briefly, for testing C1q effect on ROS-production at 30min and 2h-post treatment the monolayer cultures of hNSC were first detached into single cells, centrifuged, resuspended into Eppendorf tubes with warm DPBS$^{-/-}$, and loaded with 5µM carboxy-H2DCFDA in for 30min in RT. For ROS+ control, 100µM Rotenone (Enzo Life Sciences, ALX-350-360) were added into hNSC monolayer culture 2h prior to detachment and dye loading. For propidium iodide (PI)+ control, the cells were exposed to 1000µM H202 (Thermo Fisher Scientific, H325100) 2h prior to detachment. After centrifugation, the supernatant was removed, and the cells were resuspended into warm GM (non-treated cells and PI only controls) or GM with purified human C1q either at 0.1nM, 1nM, or 200nM concentration or temperature-inactivated C1q 200nM negative control for either 30min or 2h. Next the samples and PI+ control were stained with propidium iodide 1:1000 (Invitrogen, P3566) followed by instant analysis of median fluorescence of carboxy-H2DCFDA intensities using a BD FACSDiva 9.0 Flow Cytometer (BD Biosciences). For 24h or 48h timepoints, hNSC monolayers were first exposed to purified human C1q either at 0.1nM, 1nM, or 200nM concentration vs. non-treated and temperature-inactivated C1q controls either for 24h or 48h prior to detachment and loading of carboxy-H2DCFDA, PI staining and flow analysis. Gating for the flow cytometry analyses were set using negative control cells without carboxy-H2DCFDA, dead PI only stained cells, and ROS+ control cells with carboxy-H2DCFDA only. Time-course data was collected from one to five biologically independent experiments with one sample well per each. In all independent experiments, the cell viability was typically more than 95%. All analyses were performed by investigators blinded to experimental conditions/groups.

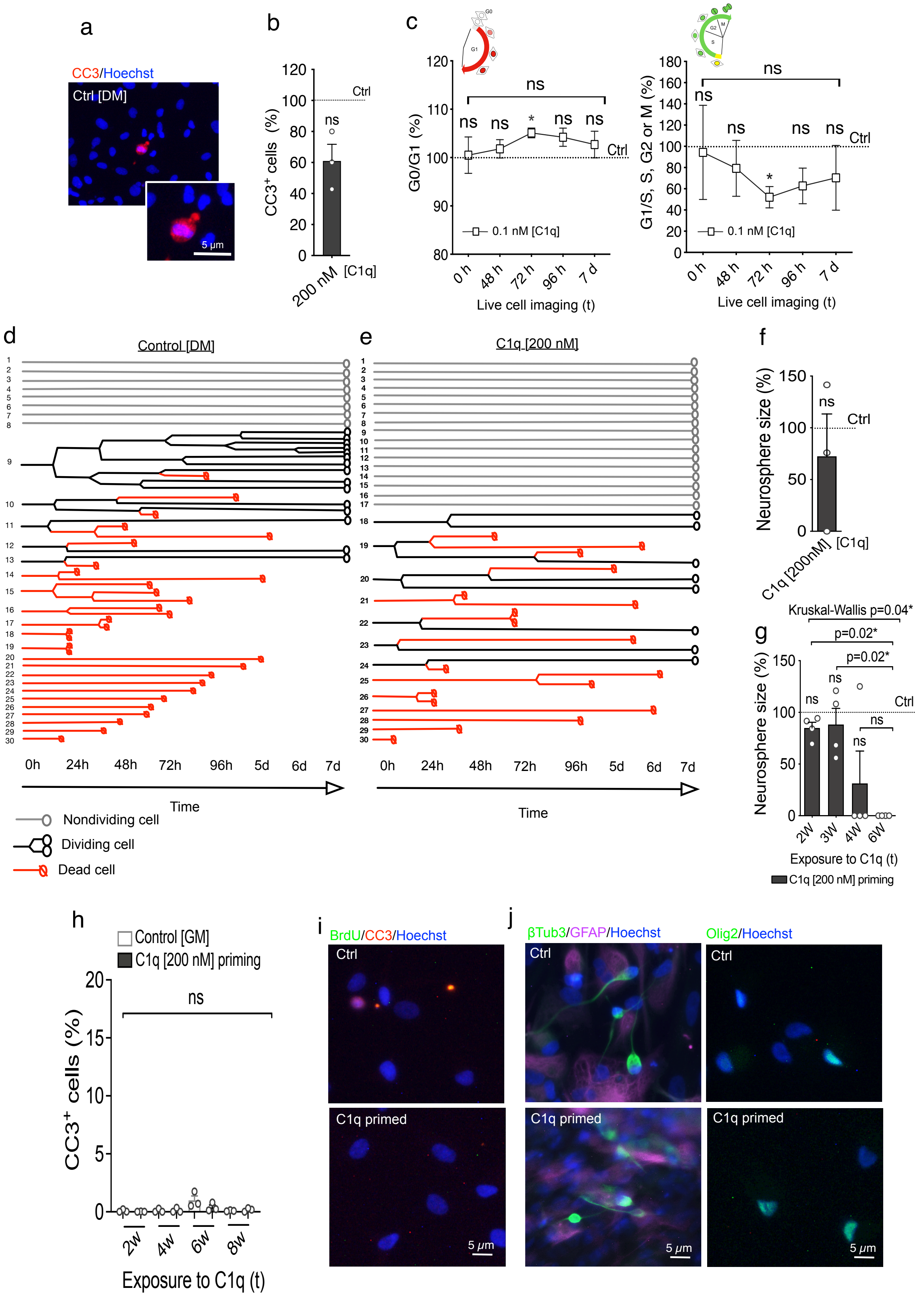
a
CC3/Hoechst
Ctrl [DM]
5 µm
b
CC3+ cells (%)
Ctrl
ns
200 nM [C1q]
c
G0/G1 (%)
ns
*
Ctrl
0.1 nM [C1q]
0 h
48 h
72 h
96 h
7 d
Live cell imaging (t)
G1/S, S, G2 or M (%)
d
Control [DM]
e
C1q [200 nM]
0h
24h
48h
72h
96h
5d
6d
7d
Time
Nondividing cell
Dividing cell
Dead cell
f
Neurosphere size (%)
C1q [200nM] [C1q]
g
Kruskal-Wallis p=0.04*
p=0.02*
2W
3W
4W
6W
Exposure to C1q (t)
C1q [200 nM] priming
h
Control [GM]
C1q [200 nM] priming
CC3+ cells (%)
8W
i
BrdU/CC3/Hoechst
Ctrl
C1q primed
5 µm
j
βTub3/GFAP/Hoechst
Olig2/Hoechst

Supplementary Fig.1: Blood plasma-derived C1q concentration promotes NSC quiescence without decline in cell survival or loss of multipotency. Related to Fig. 1.

a, An example of cleaved caspase 3 (CC3) immunocytochemistry in hNSC.

b, Quantification of CC3+ cells in C1q [200nM]-treated hNSC relative to non-treated controls (ctrl, dashed line) (*n* = 3).

c, Live-cell Fucci imaging quantification of G0/G1 (non-fluorescent/Cdt1-red) or G1/S, S, G2 and M (Geminin-green) cell cycle phases in mNSC treated with peripheral leukocyte-associated C1q concentration [0.1 nM] relative to controls (*n* = 4, total 600 cells/group). All cells in microwells were analyzed at each timepoint, with 150 cells plated/group per experiment.

d-e, Representative Fucci-mNSC lineage maps showing tracking of 30 randomly selected G1-phase cells at 0 h per group (*n* = 1), illustrating data collection for Fig.1j to compare viability between non-treated control (**d**) vs. C1q [200nM]-treated mNSC (**e**) over 7 days.

f, Neurosphere size in C1q-[200nM] treated hNSC relative to non-treated controls (*n* = 3).

g, C1q-[200nM] priming effect on neurosphere size relative to non-treated controls after medium change and analysis under normal growth conditions (*n* = 4). In absence of neurospheres, the sphere area for analysis was scored as 0.

h-i, Proportion of CC3+ cells in C1q-treated and control hNSC after extended time of C1q-priming (**h**) (*n* = 3). An example of CC3+ immunostaining in control vs. C1q [200nM]-primed hNSC (**i**).

j, hNSC sustain their multi-lineage capacity to generate bTub3+ neurons, GFAP+ astrocytes, or Olig2+ oligodendroglial cells after an extended time of C1q-priming.

Mean ± s.e.m., **b,f** 1-sample 2-tailed t-test, **c** repeated measures 1-way ANOVA and 1-sample 2-tailed t-test, **g** Kruskal-Wallis test with post hoc and 1-sample 2-tailed t-test, **h** Kruskal-Wallis test. *n* = biologically independent experiments. ns = not significant. See also Supplementary Source Data.

Supplementary Figure 2

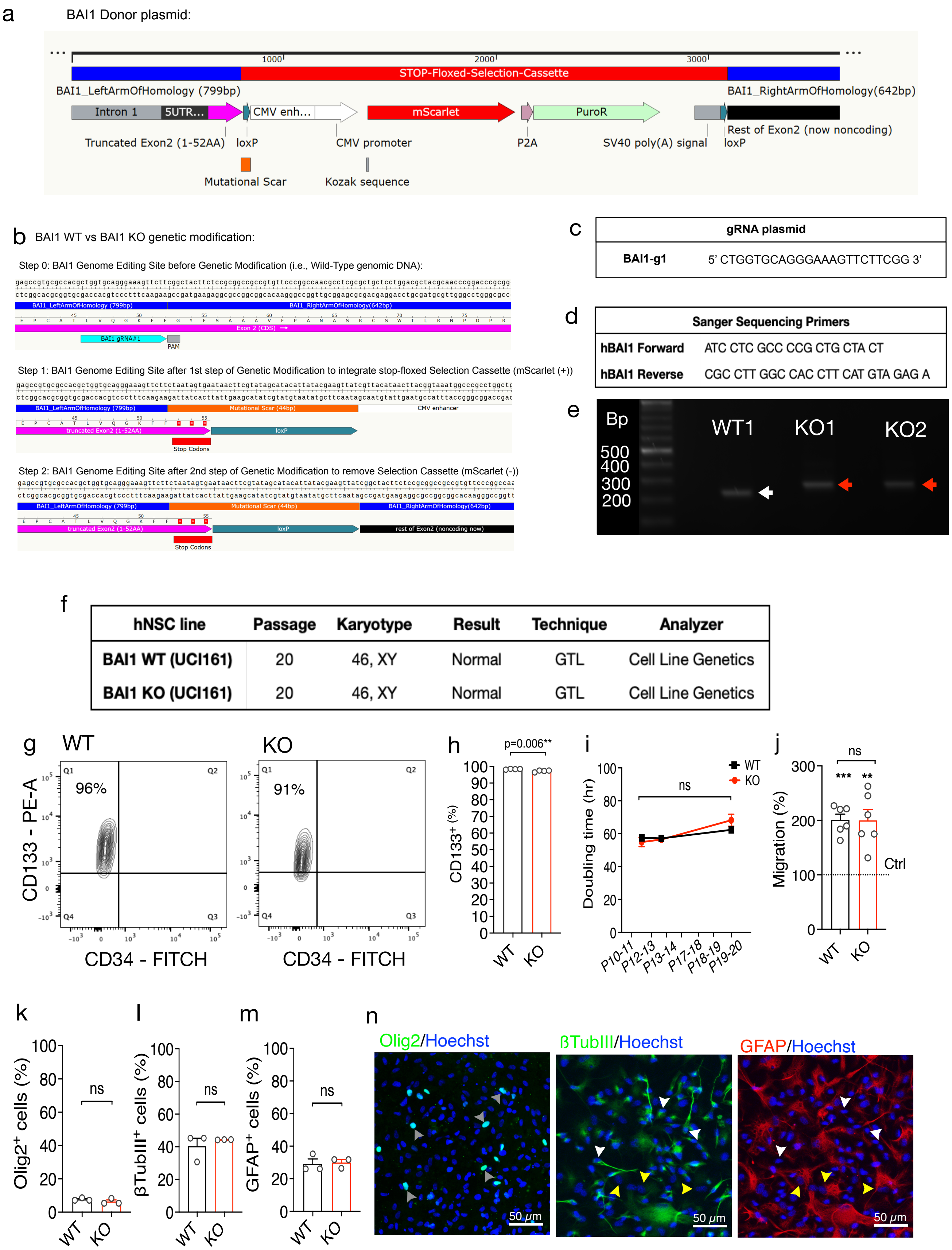

Supplementary Fig.2: WT and BAI1 KO hNSC generation; The human ADGRB1 loci (gene ID: 575) was targeted using the CRISPR/Cas9 system to create genetic knockout via homology directed repair (HDR). Related to Fig. 3.

a-b, To generate BAI1 knockouts hNSC, BAI1 donor plasmid (**a**) was co-transfected with cas9-expressing-BAI1 Guide RNA plasmid (Applied Stem Cells) to disrupt BAI1's ORF in hNSC. The genetic modification strategy entailed two rounds of genome editing (**b**).

c, BAI1 guide RNA sequence.

d, Sanger sequencing primers.

e, Homozygous insertion of the frame-shift premature stop codons in BAI1 KO hNSC was verified via PCR of genomic DNA. Insertion of this 44 bp genetic modification (red arrows in KO vs. white arrow in WT) results in the addition of 3 premature stop codons within BAI1's ORF that causes BAI1 truncation at amino acid position 52 out of 1584 amino acids total; i.e. the length of the BAI1 protein is reduced by 96.7% by the introduction of this 44 bp genetic modification, effectively creating a precise genetic knockout in hNSC.

f-n, BAI1 WT and KO hNSC exhibited sustained normal karyotype (**f**), high CD133+ stem cell proportions (**g,h**; *n* = 4), stable growth rate under *in vitro* growth conditions (**i**; *n* = 2, 3 or 4), migration response (**j**; *n* = 6), and multipotency in neural lineage differentiation (**k-n**; *n* = 3) demonstrating that both cell lines retained normal hNSC characteristics. Arrow heads: grey, Olig2+ oligodendroglial cells; white, ßTubIII+ neuronal lineage cells; yellow, GFAP+ astroglial cells.

Mean ± s.e.m, **h** unpaired 2-tailed t-test, **i** multiple unpaired t-tests, **j** unpaired 2-tailed t-test and 1-sample 2-tailed t-test, **k,l,m** 2-tailed Mann Whitney test. *n* = biologically independent experiments. ns = not significant; **p≤0.01, ***p≤0.001. Exact p-values, *n*, and uncropped gel available in Supplementary Source Data.

Supplementary Figure 3

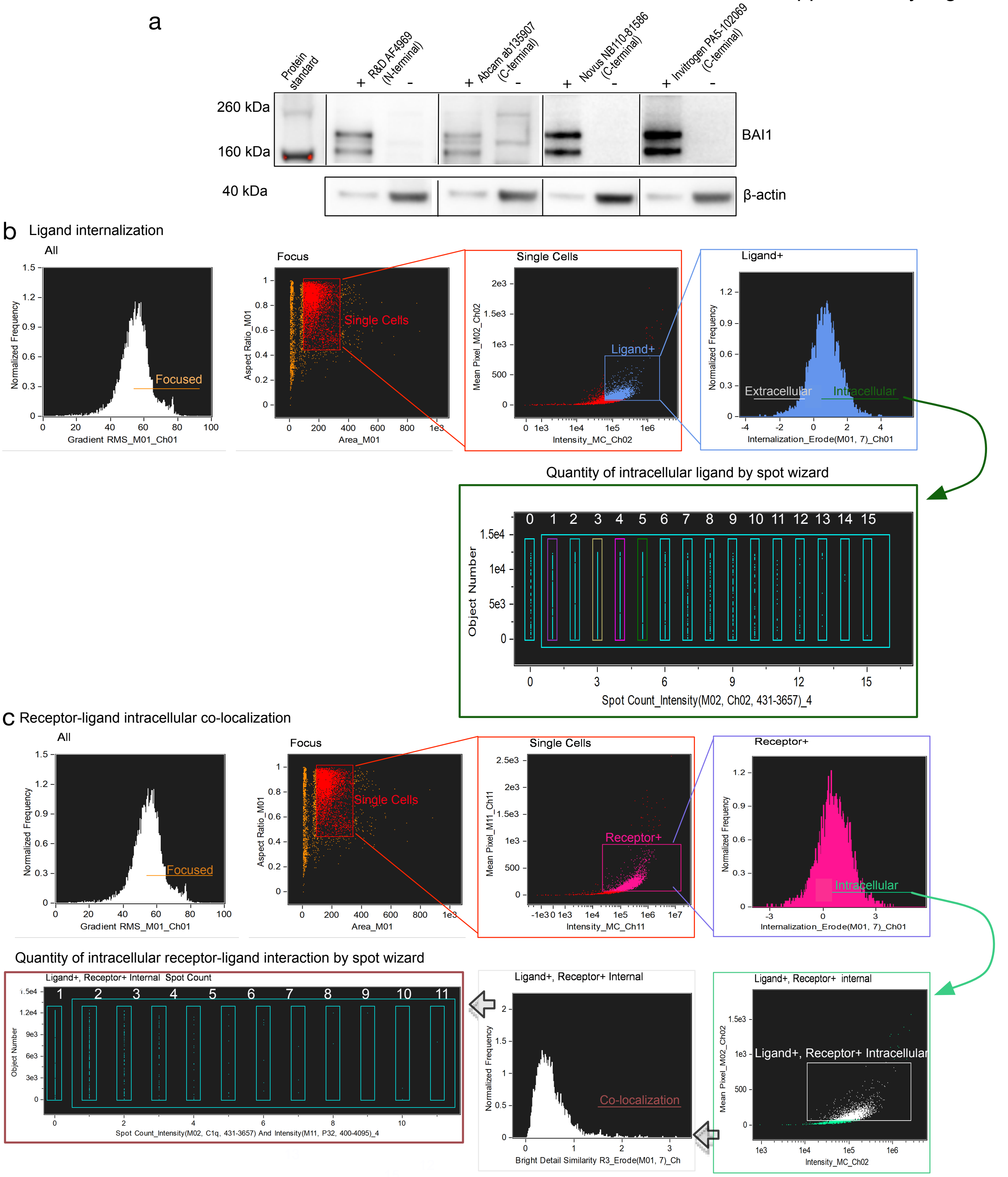

Supplementary Fig.3: BAI1 antibody testing and imagestream imaging flow cytometer gating. Related to Figs. 3, 4, and 5.

a, Commercial BAI1 antibodies generated using peptides from C-terminal vs. N-terminal region recognize full-length 174kDa BAI1 protein when analyzed using BAI1 overexpression lysate (+) vs. empty vector negative HEK293T control lysate (-) (Novus Biologicals) via Western blot under reducing conditions. Black line, non-adjacent lanes. Full blots available in Supplementary Source data.

b-c, Schematic examples of Imagestream gating for ligand internalization (**b**), receptor-ligand intracellular co-localization and spot counts (**c**) using IDEAS software. For protein internalization, a score ≥0.5 was considered internalized. A mean bright detail similarity co-localization R3 score ≥1.5 was considered colocalized. Spot count feature (see also Supplementary Fig.4o) was used for measuring the quantity of internalized protein per cell.

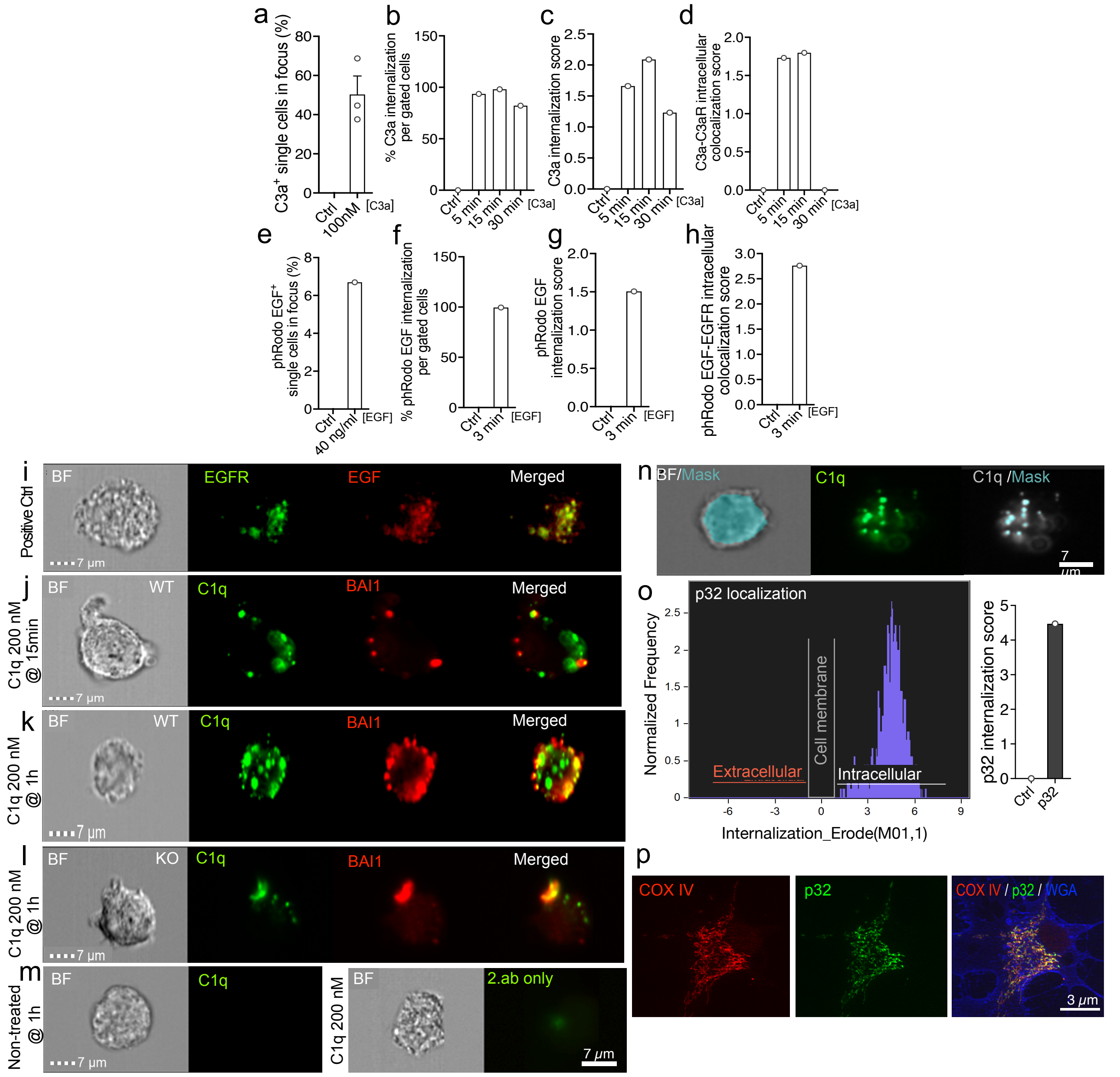

Supplementary Fig.4: Imagestream analysis validation. Related to Figs.4 and 5.

a-d, Time-course analysis of matched ligand-G protein-coupled receptor C3a-C3aR complex internalization kinetics in C3a+ in-focus single cells post-C3a treatment (*n* = 1 per timepoint; ≥7,000 cells in-focus each). **e-i**, Matched ligand-tyrosine kinase receptor EGF-EGFR complex internalization in EGF+ in-focus single cells post-phRodo EGF-treatment in hNSC (*n* = 1; 5,800 cells in-focus). An Imagestream example of pHrodo-EGF and EGFR ICC staining (**i**). For ligand/receptor internalization analysis, cells were eroded using an eroding mask of 7-pixel on a brightfield channel and cells with a ligand/receptor internalization score ≥0.5 were gated as internalized.

j-k, Time-course images of C1q-BAI1 complex internalization kinetics in BAI1+ in-focus single cells 15 min (**j**) and 1 h post-C1q treatment in BAI1 WT hNSC (**k**) captured using Imagestream (*n* = 4 per timepoint; ≥3,500 cells in-focus per each *n*).

l, C1q-BAI1 complex internalization kinetics for in-focus single cells 1h post-C1q treatment in BAI1 KO hNSC (*n* = 3; ≥3,000 cells in-focus per each *n*).

m, Representative images of negative staining controls. Non-treated, C1q-stained hNSC (left; negative control). C1q-treated, secondary antibody only (2. ab only) hNSC (right).

n, An example of Imagestream component masking for spot counts in the intracellular compartment to compare intracellular C1q quantity.

o, Imagestream analysis of p32 cellular localization and mean internalization score in non-stained vs. p32 stained hNSC based on IDEAS software.

p, Co-immunostaining of mitochondrial COX IV, p32, and plasma membrane stain wheat germ agglutinin (WGA) in hNSC. *n* = biologically independent experiments.

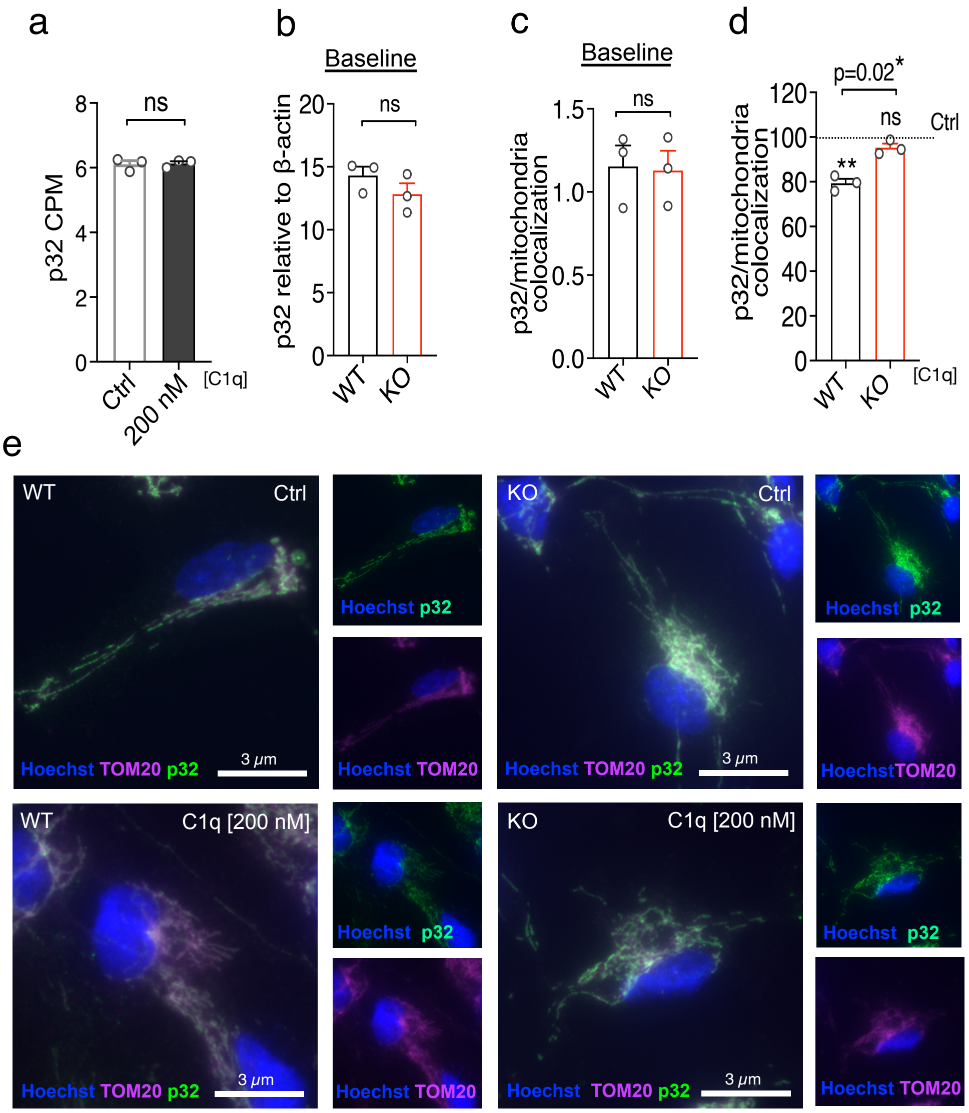

Supplementary Fig.5: p32 transcription, total p32 protein baseline, and mitochondrial p32 in hNSC. Related to Fig.4 and 5.

a, p32 transcription in non-treated control and C1q [200nM]-treated hNSC based on RNAseq. (Mean ± s.e.m counts per million reads (CPM) (*n* = 3).

b, Total p32 protein expression in non-treated BAI1 WT and KO hNSC (baseline) normalized to β-actin (*n* = 3).

c-d, Colocalization analysis of p32 in mitochondria using mitochondria membrane marker TOM20 shown as Fisher z scores in non-treated (baseline) (**c**) and C1q [200nM]-treated BAI1 WT vs. KO hNSC (**d**) (*n* = 3).

e, Example images showing co-staining of p32 and TOM20 with Hoechst nuclear stain in non-treated and C1q-treated BAI1 WT and KO hNSC. Images were captured as z-stacks of optical slices at 0.3µm intervals using a BZ-X or ApoTome 60x objective. Pearson's coefficient values were converted to z-scores using the Fisher Z-transformation function in Excel (Microsoft).

Mean ± s.e.m, **a**,**b**,**c** unpaired 2-tailed t-test, **d** unpaired 1-tailed t-test and 1-sample 2-tailed t-test. *n* = biologically independent experiments. ns = not significant; *p ≤ 0.05; ***p ≤ 0.001. Exact p-values are provided in Supplementary Source Data.

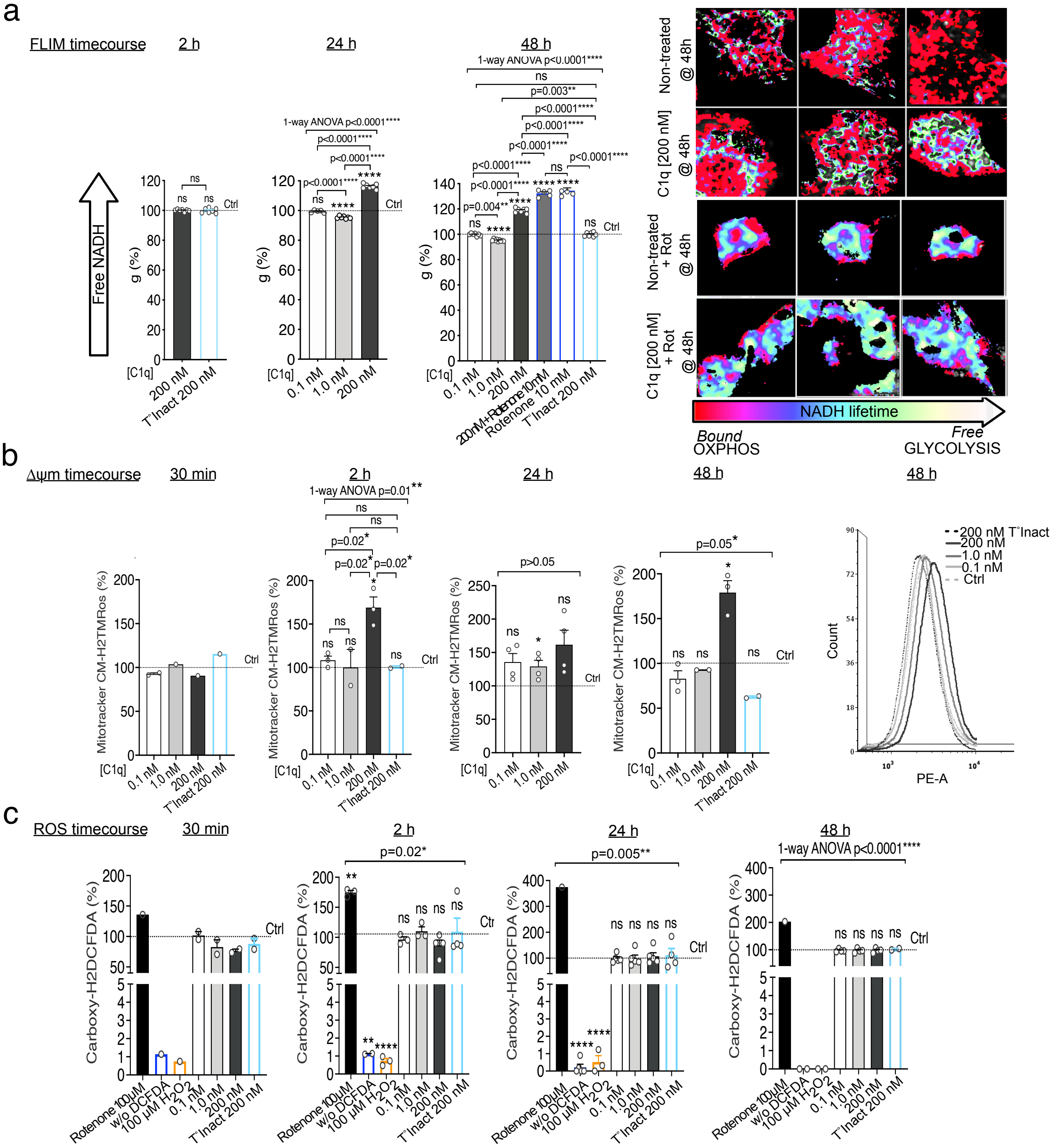

Supplementary Fig.6: Time-course analysis for NADH/NAD+ redox ratio, mitochondrial membrane potential, and ROS generation in C1q treated vs. control hNSC under normoxia. Related to Fig. 5.

a, Time-course analysis of free and bound NADH distribution using phasor two-photon FLIM in C1q-treated hNSC relative to non-treated controls (ctrl, dashed line) (*n* = 5,7 or 9 cells per group as indicated). Positive control Rotenone (Rot) 10mM-treated hNSC, and negative controls non-treated or heat-inactivated C1q [200nM] (T°Inact)-treated hNSC. Examples of two-photon FLIM images of free (red-purple)/bound (cyan-white) NADH in hNSC.

b-c, Time-course analyses of mitochondrial Δψm using Mitotracker CM-H2TMRos (**b**) and ROS generation using Carboxy-H2DCFDA in C1q-treated or C1q T°Inact-treated hNSC (negative control) relative to non-treated controls at 30min, 2h, 24h, or 48h post-C1q treatment (**c**) (*n* = 1, 2, 3, 4 or 5 as indicated). Positive control 100 μM Rotenone-treated hNSC, and negative controls non-stained live (w/o DCFDA), stained dead hNSC (H2O2 [100μM]) or C1q T°Inact-treated hNSC. Details available in Supplementary Methods.

Mean ± s.e.m, **a** unpaired 1-tailed t-test and 1-sample 2-tailed t-test, or 1-way ANOVA with post hoc test and 1-sample 2-tailed t-test, **b** 1-way ANOVA with post hoc test and 1-sample 2-tailed t-test, Kruskal-Wallis test or Welch's ANOVA with 1-sample 2-tailed t-test, **c** Kruskal-Wallis test or 1-way ANOVA with 1-sample 2-tailed t-test. *n* = biologically independent experiments. ns = not significant; *p ≤ 0.05; **p ≤ 0.01; ***p ≤ 0.001; ****p ≤ 0.0001: Exact *n* and p-values provided in Supplementary Source Data.

Supplementary Figure 7

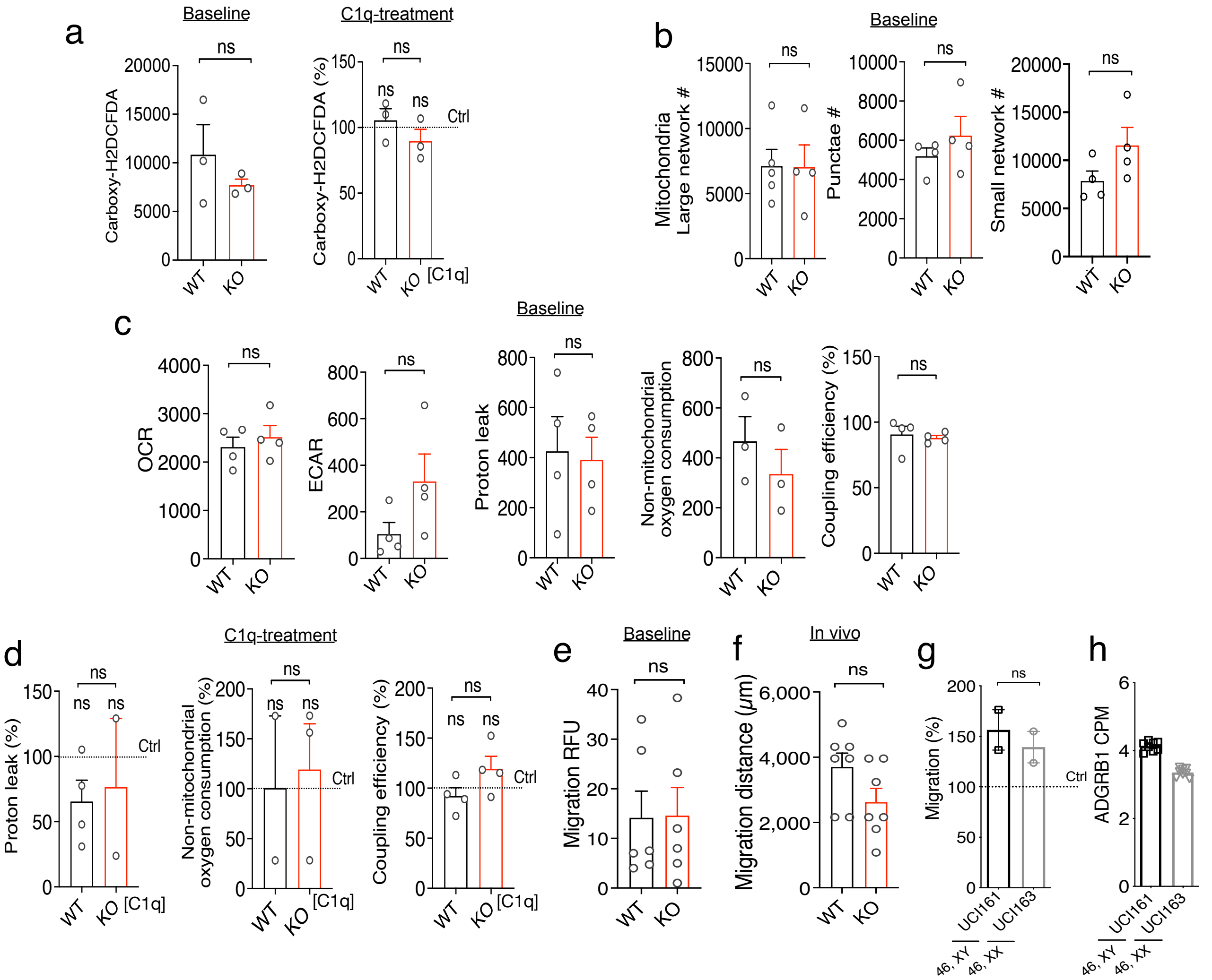

Supplementary Fig.7: ROS production, bioenergetics, mitochondria morphology, and migration capacity in BAI1 WT and KO hNSC. Related to Figs. 5 and 7.

a, ROS production and bioenergetics in non-treated (baseline) vs. C1q [200nM]-treated BAI1 WT and KO hNSC (*n* = 3).

b, Mitochondria morphology in non-treated (baseline) BAI1 WT and KO hNSC (*n* = 4 or 5 as indicated).

c,d, Bioenergetics in non-treated (baseline) (**c**) vs. C1q [200nM]-treated BAI1 WT and KO hNSC (**d**) (*n* = 2, 3 or 4 as indicated).

e, *In vitro* migration capacity (chemoattraction) in non-treated BAI1 WT and KO hNSC (baseline) measured using transwell assays (*n* = 6).

f, Maximal distance of BAI1 WT and KO hNSC migration (cell spread rostral and caudal relative to the injury epicenter) at 2 weeks post-transplantation in vivo was determined by the distance between first and last spinal cord section with STEM121+ cells visualized using an inverted microscope (IX71, Olympus) (*n* = 7 mice/group).

g-h, Both male and female hNSC lines respond to C1q [200mM] treatment (*n* = 2, 4 transwells each) (**g**) and express BAI1 in RNAseq (*n* = 8) (**h**). Dashed line, non-treated control of each hNSC line.

Mean ± s.e.m., **a,b,f** unpaired 2-tailed t-test, **c** unpaired 2-tailed t-test, or 1-tailed Mann Whitney test, **d** 1-tailed Mann Whitney test or unpaired 1-tailed t-test with 1-sample 2-tailed t-test, **e** 1-tailed Mann Whitney test, **g** unpaired 2-tailed t-test. *n* = biologically independent experiments. ns = not significant; *p ≤ 0.05, **p ≤ 0.01: Exact *n* and p-values provided in Supplementary Source Data.

Supplementary Table 1: hNSC do not express C1q or lytic complement pathway (C5-C9) transcripts, but do express C1R and C1S, as well as Frizzled receptors and LRP co-receptors based on bulk RNAseq (*n* = 3 biologically independent experiments). Data is shown as average fragments per kilobase of exon per million mapped fragments (FPKM) ± standard deviation. Related to Fig. 1.

Symbol	Name	HGNC ID	Average (N1-3)	Standard deviation (N1-3)
**FZD1**	Frizzled Class Receptor 1	HGNC: 4038	1719	203
**FZD2**	Frizzled Class Receptor 2	HGNC: 4040	885	185
**FZD4**	Frizzled Class Receptor 4	HGNC: 4042	518	13
**FZD7**	Frizzled Class Receptor 7	HGNC: 4045	316	43
**FZD8**	Frizzled Class Receptor 8	HGNC: 4046	2272	442
**LRP5**	LDL Receptor Related Protein 5	HGNC: 6697	1920	266
**LRP6**	LDL Receptor Related Protein 6	HGNC: 6698	3667	542
**C1QA**	complement C1q A chain	HGNC:1241	0	0
**C1QB**	complement C1q B chain	HGNC:1242	0	0
**C1QC**	complement C1q C chain	HGNC:1245	0	0
**C1R**	complement C1r	HGNC:1246	3498	492
**C1S**	complement C1s	HGNC:1247	2222	672
**C5**	complement C5	HGNC:1331	472	75
**C6**	complement C6	HGNC:1339	0	1
**C7**	complement C7	HGNC:1346	0	0
**C8A**	complement C8 alpha chain	HGNC:1352	0	0
**C8B**	complement C8 beta chain	HGNC:1353	0	0
**C8G**	complement C8 gamma chain	HGNC:1354	6	4
**C9**	complement C9	HGNC:1358	0	0

Supplementary Table 2: Subcellular enrichment analysis for compartments associated with receptor-mediated endocytic trafficking by identified prey proteins. These data represent unbiased nanoLC-MS/MS of pull-down experiments in hNSC using internalized C1q as a bait. A FDR p ≤ 0.05 was considered significant (*n* = 4 biologically independent experiments). Related to Fig. 4.

Compartment	p	FDR	n	Proteins
Ribosome	1.24E-42	1.73E-41	42	MRPL14, ABCF1, RPL15, RPL27, RPS7, RPS9, RPS10, RPS23, RPS27, RPL23, RPS6, RPS20, RPS21, RPS29, RPL3, RPL4, RPL5, RPL6, RPL7, RPL7A, RPL8, RPL10, RPL17, RPL18A, RPL19, RPL24, RPL26, RPL30, RPL28, RPL31, RPL32, RPL34, RPL35A, RPL36AL, RPL14, RPL35, RPL36, RPSA, RPS2, RPS15, RPS15A, BTF3
Extracellular region	2.38E-28	3.09E-27	113	A1BG, ACTN4, AEBP1, APOE, APP, C1QA, C4B, C4BPA, CALR, CLU, COL12A1, VCAN, NCAN, CTSD, DDX3X, DYNC1H1, FABP5, FBN2, FGF2, XRCC6, HP, HPX, HSPA1B, TNC, IGFBP3, CCN1, ILF2, LAMA5, LAMB1, LAMC1, LGALS3, MFGE8, NID1, YBX1, P4HB, PA2G4, PF4, HTRA1, SDCBP, SRP14, TGFBI, PXDN, NRP2, SLIT2, NTN1, ACTR2, EMILIN1, PDAP1, SCCPDH, CEMIP, ADAMTS16, LAMA1, AGRN, SERPINH1, CNP, HLA-DRB1, IGHM, MMP14, PGK1, RDX, PRDX5, GLIPR2, AHCY, ATP1A1, CAPN5, CCT6A, CD44, CTNNB1, EIF2S1, EIF2S3, FASN, FAT1, FLOT2, GLG1, GNAI2, RPSA, LDHB, MEST, MYH9, MYL6, SLC25A3, RAC1, RPL3, RPL4, RPL5, RPL24, RPL26, RPL27, RPL30, RPL28, RPL31, RPL34, RPL35A, RPS2, RPS9, RPS15A, RPS20, RPS29, SLC25A1, STAU1, TCP1, CCT3, MOGS, RAB7A, EIF3B, IQGAP1, RPL14, RPL23, PDCD6IP, ALYREF, FLOT1, SND1, MARCKSL1
Cytoplasm	6.21E-19	7.45E-18	164	GLG1, HLA-DRB1, RAC1, GLIPR2, RAB7A, DDX6, CAPRIN1, CALR, TCP1, VDAC2, FLOT2, MFGE8, ACTN4, AEBP1, APOE, APP, CAPN5, CAMK2D, CCT6A, CLU, CNP, CTNNB1, DDX1, DDX3X, EIF2S3, ETF1, FABP5, FASN, FGF2, GNAI2, HSPA1B, RPSA, LDHB, LGALS3, MAP2, MMP14, MYH9, NACA, YBX1, PA2G4, PF4, RPL3, RPL4, RPL5, RPL7, RPL7A, RPL24, RPL26, RPS9, RPS15A, SDCBP, SRP14, STAU1, EIF3A, IQGAP1, EIF2S2, RPL23, SLIT2, NTN1, MAP4K4, ACTR2, G3BP1, ALYREF, PRDX5, RPL36, CEMIP, MARCKSL1, MTDH, CPT1A, ECI1, NDUFS3, SLC25A3, SLC25A13, SCCPDH, MRPL14, SLC25A1, CTSD, FLOT1, ATP1A1, SRPX, SERPINH1, MEST, P4HB, RPL10, RPL34, RPL36AL, RPN1, RPS6, RPS23, PXDN, MOGS, HNRNPR, SYNCRIP, SEC61B, HSD17B12, HACD3, LRRC59, LEMD2, SEC61A1, COL12A1, VCAN, TNC, IGFBP3, CCN1, LAMB1, LAMC1, RPL6, RPS21, CD44, NCAN, AGRN, TGFBI, ABCF1, AP2A1, AP2B1, AHCY, BTF3, CALD1, DYNC1H1, EIF2S1, XRCC6, MYL6, PGK1, PRKDC, HTRA1, RPL8, RPL15, RPL17, RPL18A, RPL19, RPL27, RPL30, RPL28, RPL31, RPL32, RPL35A, RPS2, RPS7, RPS10, RPS15, RPS20, RPS27, RPS29, CCT3, EIF3B, EIF3C, EIF3F, RPL14, PDCD6IP, RPL35, PDAP1, SND1, EIF3L, DPYSL5, CCDC124, RDX, A1BG, GRIK3, HNRNPU, HP, ILF2, FAT1, FUS, HPX
Endoplasmic reticulum	8.46E-11	9.31E-10	49	APOE, APP, ATP1A1, CALR, SERPINH1, HSPA1B, MEST, P4HB, RPL5, RPL10, RPL24, RPL34, RPL36AL, RPN1, RPS6, RPS23, STAU1, PXDN, MOGS, SRPX, HNRNPR, SYNCRIP, SEC61B, HSD17B12, HACD3, LRRC59, CEMIP, MTDH, LEMD2, SEC61A1, COL12A1, VCAN, TNC, IGFBP3, CCN1, LAMB1, LAMC1, MFGE8, RAC1, SDCBP, RPL4, RPL6, RPS21, CAMK2D, PDCD6IP, HLA-DRB1, RPL27, RPS29, CLU
Intracellular vesicle	2.32E-07	2.32E-06	48	CALR, TCP1, VDAC2, FLOT2, MFGE8, APP, ATP1A1, FLOT1, APOE, RAB7A, CTSD, HLA-DRB1, AP2A1, AP2B1, CEMIP, CAMK2D, CD44, FABP5, LGALS3, RAC1, IQGAP1, A1BG, ACTN4, CLU, PF4, SCCPDH, MMP14, PRDX5, DDX3X, XRCC6, SRP14, DYNC1H1, PA2G4, SDCBP, ACTR2, HP, ILF2, AHCY, CNP, FASN, P4HB, RPN1, PDCD6IP, SND1, MYH9, HPX, HSPA1B, PDAP1
Lysosome	0.002	0.01	15	CALR, CTSD, RAB7A, HLA-DRB1, FLOT1, DYNC1H1, FABP5, PA2G4, SDCBP, ACTR2, AP2A1, AP2B1, VCAN, NCAN, AGRN
Vacuole	0.002	0.02	16	RAB7A, CALR, CTSD, HLA-DRB1, FLOT1, SRPX, DYNC1H1, FABP5, PA2G4, SDCBP, ACTR2, AP2A1, AP2B1, VCAN, NCAN, AGRN
Cytoskeleton	0.004	0.03	34	TCP1, CTNNB1, HNRNPU, DDX6, ACTN4, MYH9, DDX3X, DYNC1H1, GNAI2, HSPA1B, RPS7, PDCD6IP, FLOT1, CCDC124, CALD1, CLU, GLG1, P4HB, RAC1, SDCBP, CCT3, MARCKSL1, CCT6A, MAP2, EIF3A, IQGAP1, STAU1, HLA-DRB1, ACTR2, MYL6, FLOT2, RDX, APP, MMP14
