# Supplementary material for: C1q drives neural stem cell quiescence by regulating cell cycle and metabolism through BAI1
Source: Nat Commun. 2025 Dec 11;16:11386. doi: 10.1038/s41467-025-66185-x (PMC12738564; doi:10.1038/s41467-025-66185-x)
Supplement: Supplementary file 2 — Reporting Summary [file 41467_2025_66185_MOESM2_ESM.pdf]

nature portfolio

Corresponding author(s): Piltti KM and Anderson AJ

Last updated by author(s): Oct 28, 2025

Reporting Summary

Nature Portfolio wishes to improve the reproducibility of the work that we publish. This form provides structure for consistency and transparency in reporting. For further information on Nature Portfolio policies, see our Editorial Policies and the Editorial Policy Checklist.

Statistics

For all statistical analyses, confirm that the following items are present in the figure legend, table legend, main text, or Methods section.

n/a	Confirmed	
☐	☒	The exact sample size (*n*) for each experimental group/condition, given as a discrete number and unit of measurement
☐	☒	A statement on whether measurements were taken from distinct samples or whether the same sample was measured repeatedly
☐	☒	The statistical test(s) used AND whether they are one- or two-sided<br>*Only common tests should be described solely by name; describe more complex techniques in the Methods section.*
☐	☒	A description of all covariates tested
☐	☒	A description of any assumptions or corrections, such as tests of normality and adjustment for multiple comparisons
☐	☒	A full description of the statistical parameters including central tendency (e.g. means) or other basic estimates (e.g. regression coefficient) AND variation (e.g. standard deviation) or associated estimates of uncertainty (e.g. confidence intervals)
☐	☒	For null hypothesis testing, the test statistic (e.g. *F*, *t*, *r*) with confidence intervals, effect sizes, degrees of freedom and *P* value noted<br>*Give P values as exact values whenever suitable.*
☒	☐	For Bayesian analysis, information on the choice of priors and Markov chain Monte Carlo settings
☒	☐	For hierarchical and complex designs, identification of the appropriate level for tests and full reporting of outcomes
☒	☐	Estimates of effect sizes (e.g. Cohen's *d*, Pearson's *r*), indicating how they were calculated

Our web collection on statistics for biologists contains articles on many of the points above.

Software and code

Policy information about availability of computer code

Data collection	Illumina HiSeq 4000 Sequencing System (Illumina), BD Fusion Cell Sorter (BD Biosciences), UltiMate 3000 RSLC system (Thermo Fisher Scientific) coupled in-line to an Orbitrap Fusion Lumos mass spectrometer (Thermo Fisher Scientific), Amnis® ImageStream®x Mark II Imaging Flow Cytometer (Luminex), BD FACSDiva 9.0 Flow Cytometer (BD Biosciences).
Data analysis	IDEAS software version 6.2, FlowJo version 10.10.0. (TreeStar) or FCS Express version 6 (De Novo Software), Imaris software 7.5.2. (Andor Technology Ltd), Prism version 10 (GraphPad), MaxQuant version 1.6.0.16 (Max-Planck-Institute), Differential Enrichment analysis of Proteomics data (DEP) version 1.28 (Bioconductor), FastQC (Babraham Institute), SortMeRNA (LIFL), Trimmomatic (Max Planck Institute), EdgeR (Bioconductor), Gene Set Enrichment Analysis (GSEA) and Molecular Signatures Database (MSigDB) (Broad Institute), SimFCS software (Laboratory for Fluorescence Dynamics, UC Irvine), CellProfiler version 4.2.1 (CellProfiler), MitoMo Integrated Mitochondrial image analysis software (Visualization and Intelligent Systems Laboratory, UC Riverside), Matlab (MathWorks), Agilent Seahorse Wave version 2.6.1 (Agilent Technologies), Prism version 10 (GraphPad).

For manuscripts utilizing custom algorithms or software that are central to the research but not yet described in published literature, software must be made available to editors and reviewers. We strongly encourage code deposition in a community repository (e.g. GitHub). See the Nature Portfolio guidelines for submitting code & software for further information.

Data

Policy information about availability of data

All manuscripts must include a data availability statement. This statement should provide the following information, where applicable:

- Accession codes, unique identifiers, or web links for publicly available datasets
- A description of any restrictions on data availability
- For clinical datasets or third party data, please ensure that the statement adheres to our policy

The main data supporting the results of this study are available within the Manuscript, Supplementary data, and Source data.

Research involving human participants, their data, or biological material

Policy information about studies with human participants or human data. See also policy information about sex, gender (identity/presentation), and sexual orientation and race, ethnicity and racism.

Reporting on sex and gender	No human participants were involved in this study.<br><br>Two different multipotent human male fetal CNS-derived stem cell lines (referred to as hNSC) were used in this study; Stem Cells Inc. 2491.2 (PMID: 11121071) and UCI161 hNSC (UC Irvine, Anderson AJ) were isolated from human fetal brain at gestational week 16 to 20 with comparable derivation methods and enriched for CD133 stem cell marker (PMID: 11121071, PMID: 12205691). All human stem cell lines evaluated responded to C1q treatment with a decrease in cell proliferation. While sex-based differences between NSC lines were not compared in this study, both male and female hNSC lines responded to C1q [200mM] treatment (Supplementary Fig.7g), and expressed BAI1 in RNAseq (Supplementary Fig.7h).
Reporting on race, ethnicity, or other socially relevant groupings	No human participants were involved in this study.
Population characteristics	No human participants were involved in this study.<br><br>Multipotent human neural stem cell line, UCI161 was established in the Anderson laboratory at UCI from male fetal brain tissue procured at gestational 16 weeks under UCI hSCRO (#3682) and IRB (BUA-R120) approval. These cells were derived prior to June 5, 2019 (NIH NOT-OD-19-128). BAI1 wildtype (WT) and knockout (KO) hNSC (UC Irvine, Anderson AJ) were generated using CRISPR Cas9 gene editing of UCI161. Multipotent human neural stem cell line, 2491.2 was established by the StemCells Inc. from male fetal brain tissue procured between gestational weeks 16–20 as previously described (PMID: 11121071). Comparable derivation methods and CD133 stem cell marker enrichment were employed in generation of UCI161 and 2491.2 hNSC lines, both of which exhibit neurosphere-initiating capacity, respond to chemotactic cues, and retain multipotency for over 20 passages generating neurons, oligodendrocytes, and astrocytes (PMID: 11121071,PMID: 12205691).
Recruitment	No human participants were involved in this study.
Ethics oversight	No human participants were involved in this study.<br><br>All experimental paradigms using human stem cells were conducted in accordance with the Human Stem Cell Research Oversight (hSCRO; #3682) committee and Institutional Biosafety committee (IBC; BUA-R120) at UCI. Human stem cell lines were derived prior to June 5, 2019 (NIH NOT-OD-19-128). Informed consent was obtained from all donors of human fetal brain tissue for establishing the stem cell lines used in this study.

Note that full information on the approval of the study protocol must also be provided in the manuscript.

Field-specific reporting

Please select the one below that is the best fit for your research. If you are not sure, read the appropriate sections before making your selection.

[x] Life sciences [ ] Behavioural & social sciences [ ] Ecological, evolutionary & environmental sciences

For a reference copy of the document with all sections, see nature.com/documents/nr-reporting-summary-flat.pdf

Life sciences study design

All studies must disclose on these points even when the disclosure is negative.

Sample size	No statistical tools were used for predetermine sample sizes for this manuscript. All in vitro cell culture data for each studied condition in this manuscript has been replicated across multiple biologically independent experiments on different days or timepoints (timecourse analysis). Number of biologically independent replicates or measurements referring to numbers each experiment was repeated independently with similar results are listed in the Figure legends and in the Source Data. Number of technical replicates included are listed in the Methods. Because BAI1 KO hNSC line engraftment has not been previously quantified in the SCI mouse model, the number of mice to test histological enraftment of BAI1 hNSC in vivo was determined based on published historical data.<br>Statistical analysis methodology including corresponding details can be found both within the Figure Legends and in the Source Data. All data

	are presented as mean±standard mean of error (s.e.m). Before conducting statistical analyses, the data were assessed for normality and homogeneity of variances, and appropriate parametric or non-parametric tests were applied as outlined in the Source Data or Supplementary Source Data.
Data exclusions	No animals or tissue sections immunostained in 1/12 sampling sequence were excluded from histological or statistical analysis. For in vitro assays, exclusions due to technical errors during sample collection or processing are described in the Methods and Source Data. In addition, ROUT or Grubb's tests were used to identify outliers in the data using Prism version 10 (GraphPad) as also described in the Methods and Source data.
Replication	Number of biologically independent replicates or measurements referring to numbers each experiment was repeated independently with similar results are listed in the Figure legends and in the Source Data. The number of technical replicates within each independent experiment are listed in the Methods.
Randomization	Assignment of animals, cell culture vessels or wells into experimental groups as well as order of groups for data collection and analysis were randomized throughout the study. This included ensuring that treatments applied to culture wells were distributed to different well locations, that section sampling for stereology was randomized across sectioning block, etc.
Blinding	All experiments including animal care, histological analysis, image acquisition, or data collection and analysis were conducted by investigators blinded to experimental conditions/groups as described in the Methods. Blinding was performed using a coded system and random group assignment. Code breaking for each experimental task was done after all the analyses were completed.

Reporting for specific materials, systems and methods

We require information from authors about some types of materials, experimental systems and methods used in many studies. Here, indicate whether each material, system or method listed is relevant to your study. If you are not sure if a list item applies to your research, read the appropriate section before selecting a response.

Materials & experimental systems

n/a	Involved in the study
☐	☒ Antibodies
☐	☒ Eukaryotic cell lines
☒	☐ Palaeontology and archaeology
☐	☒ Animals and other organisms
☒	☐ Clinical data
☒	☐ Dual use research of concern
☒	☐ Plants

Methods

n/a	Involved in the study
☒	☐ ChIP-seq
☐	☒ Flow cytometry
☒	☐ MRI-based neuroimaging

Antibodies

Antibodies used	All antibodies used in this study including the manufacture and catalog numbers and dilutions used are listed in the Methods and Supplementary Methods.
Validation	All antibodies used in this study have been used is several previously published studies. BAI1 primary antibodies used in this study were validated using Western blots and confirming that they all recognize full-length 174kDa BAI1 protein band in BAI1 overexpression lysate (+) vs. empty vector negative HEK293T control lysate (-) (Novus Biologicals) (Supplementary Fig. 3a) as well as in BAI1 WT hNSC lysate vs. BAI1 WT hNSC lysate (Fig. 3b). All other primary antibodies have been previously validated in in Western blots and/or immunostaining via primary/secondary omission controls or via use of multiple antibodies directed against the same protein using either western blotting, immunocytochemistry or flow cytometry. Specificity of secondary antibodies was validated by confirming the lack of signal in secondary-only (primary antibody omission) stained controls.

Eukaryotic cell lines

Policy information about cell lines and Sex and Gender in Research

Cell line source(s)	Multipotent human neural stem cell line, UCI161 was established in the Anderson laboratory at UCI from male fetal brain tissue procured at gestational 16 weeks under UCI hSCRO and IRB approval. These cells were derived prior to June 5, 2019 (NIH NOT-OD-19-128). BAI1 wildtype (WT) and knockout (KO) hNSC (UC Irvine, Anderson AJ) were generated using CRISPR Cas9 gene editing of UCI161. Multipotent human neural stem cell line, 2491.2 was established by the StemCells Inc. from male fetal brain tissue procured between gestational weeks 16–20 as previously described (PMID: 11121071). Comparable derivation methods and CD133 stem cell marker enrichment were employed in generation of UCI161 and 2491.2 hNSC lines, both of which exhibit neurosphere-initiating capacity, respond to chemotactic cues, and retain multipotency for over 20 passages generating neurons, oligodendrocytes, and astrocytes (PMID: 11121071,PMID: 12205691). Multipotent mouse NSC line was derived from single fluorescent ubiquitination-based cell-cycle indicator (Fucci) mouse (PMID: 18267078, PMID: 24786503) embryo cortices at E11 (referred to as Fucci-mNSC) in the Anderson laboratory at UCI as previously described (PMID: 2905082). Each of these human and mouse stem cell lines shared the identified C1q effect on cell proliferation.
Authentication	Multipotent human neural stem cell lines, UCI161 and 2491.2 have been used in multiple studies in our research group, including in previous publications (PMID: 28687659, PMID: 32894219, PMID: 38499577). Both hNSC lines were generated

from tissue samples procured at comparable developmental stages utilizing equivalent methods to enrich for CD133 stem cell marker (PMID: 11121071). Both cell lines have been screened for normal karyotype and confirmed to be negative for adventitious agents. In addition, we have verified neurosphere-initiating capacity, chemotactic response, and multipotency for neural lineage differentiation in both human stem cell lines (PMID: 11121071,PMID: 12205691). All stem cell lines generated in our group have been banked for use at low passage number (≤20), and also are routinely tested for normal karyotype.
BAI1 wildtype (WT) and knockout (KO) hNSC were generated using CRISPR Cas9 gene editing of hNSC line UCI161. Stable loss of total BAI1 protein expression was confirmed by Western blotting, and functional-level, by performing PLA specific for the protein-protein interaction of BAI1 with its ligand, C1q, in both BAI1 KO and WT cells. Two different BAI1 KO lines were generated with the same method. No substantial differences between the cell lines generated were detected regarding their CD133+ content, multipotency, migration, or proliferative capacity. Follow-up experiments in vitro were done only for the transplanted cell line with at least independent biological experimental triplicates. BAI1 WT and BAI1 KO hNSC exhibited sustained normal karyotype, high CD133+ stem cell proportions, stable growth rate under in vitro growth conditions, migration response, and multipotency in neural lineage differentiation demonstrating that both cell lines retained normal hNSC characteristics.
Multipotent mouse Fucci-expressing NSC line derived from mouse embryo cortices at E11 has been used in multiple studies in our group including in previous publication (PMID: 2905082). We have validated that Fucci-mNSC retain neurosphere-initiating capacity, chemotactic response, and multipotency for neural lineage differentiation. Each of these human and mouse NSC lines used in this study share the identified C1q effect on cell proliferation and migration.

Mycoplasma contamination

All cell lines used in this study were routinely tested for Mycoplasma contamination and verified to be mycoplasma-free before using them in any in vitro and in vivo experiments.

Commonly misidentified lines
(See ICLAC register)

n/a

Animals and other research organisms

Policy information about studies involving animals; ARRIVE guidelines recommended for reporting animal research, and Sex and Gender in Research

Laboratory animals

10 week old immunodeficient female Rag-1- mice (n=14; B6.12957-Rag1tm1Mom/J; The Jackson Laboratory, 002216) were used as a SCI model for testing biological significance of BAI1 for in hNSC in vivo. Sex was not considered in the study design: A female model was selected to avoid bladder complications and urolithiasis that frequently occur in male mice following SCI (PMID: 19831737, PMID: 19091977).

Wild animals

No wild animals were used in this study.

Reporting on sex

A female mouse model was selected to avoid bladder complications and urolithiasis that frequently occur in male mice following SCI (PMID: 19831737, PMID: 19091977).

Sex was not considered in all elements of in vitro analysis of human cells because of the volume of data collected, however, data addressing this issue has been included to the manuscript. Briefly, while sex-based differences between NSC lines were not specifically compared, we report that both male and female hNSC lines responded to C1q [200mM] treatment (Supplementary Fig.7g), and expressed BAI1 in RNAseq (Supplementary Fig.7h). Two different multipotent human male fetal CNS-derived stem cell lines (referred to as hNSC) were used in this study; Stem Cells Inc. 2491.2 (PMID: 11121071) and UCI161 hNSC (UC Irvine, Anderson AJ) were isolated from human fetal brain at gestational week 16 to 20 with comparable derivation methods and enriched for CD133 stem cell marker (PMID: 11121071, PMID: 12205691). Both UCI161 and 2491.2 lines have been used in multiple studies in our research group, including in previous publications (PMID: 28687659, PMID: 32894219, PMID: 38499577). Both human stem cell lines used in this study shared the identified C1q effect on cell proliferation.

Field-collected samples

No field-collected samples were used in this study.

Ethics oversight

Animal care, and data analysis were performed by investigators blinded to the experimental groups. All animal housing conditions, procedures, and animal care were approved by the UCI Institutional Animal Care and Use Committee (IACUC; AUP-23-023). All animals were housed in cages with Alpha-dri bedding, nestlets, and housing accordance with the IACUC guidelines on a 12h dark/light cycle with food and water ad libitum. Derivation and usage of human neural stem cell line UCI161 (BAI1 WT and BAI1 KO hNSC) for all in vitro and in vivo work was reviewed and approved by the UCI human Stem Cell Research Oversight Comittee (hSCRO; #3682) and Institutional Biosafety committee (IBC; BUA-R120).

Note that full information on the approval of the study protocol must also be provided in the manuscript.

Plants

Seed stocks	No plants were used in this study.
Novel plant genotypes	n/a
Authentication	n/a

Flow Cytometry

Plots

Confirm that:

- [x] The axis labels state the marker and fluorochrome used (e.g. CD4-FITC).
- [x] The axis scales are clearly visible. Include numbers along axes only for bottom left plot of group (a 'group' is an analysis of identical markers).
- [x] All plots are contour plots with outliers or pseudocolor plots.
- [x] A numerical value for number of cells or percentage (with statistics) is provided.

Methodology

Sample preparation

For imaging flow cytometry, hNSC monolayer cultures at 70-80% confluence were washed with DPBS-/- and detached using Trypsin/EDTA (Cell Applications) or non-enzymatic cell dissociation solution (Sigma-Aldrich). Cells were aliquoted and fixed using 2% PFA for 15min on ice followed by wash with 1% fish gelatin (Sigma Aldrich, G7041) in 0.1M TBS, blocking, and 1 minute permeabilization using DPBS supplemented with 0.1% or 0.02% Triton X-100, 5% goat or donkey serum (Jackson Immunoresearch). Cells were labelled with either pHrodo™ red Epidermal Growth Factor (EGF) Conjugate (Thermo Fisher Scientific, P35374), CellLight™ Early Endosomes-RFP, BacMam 2.0 (Invitrogen, C10587), or CellLight™ Golgi-RFP, BacMam 2.0 (Invitrogen, C10593) or primary antibodies raised against anti-C1q (1:50, Abcam, Ab71940), anti-human BAI1 (1:50, R&D systems, AF4969), anti-BAI1 (1:50, Lifespan Biosciences, LS-C120632), anti-human complement C3a (1:50, Millipore/ Chemicon, CBL191), anti-C3aR (1:50, Abcam, Ab103629), anti-EGFR (Abcam, EP38y, ab52894), recombinant anti-GC1qR (1:50, Abcam, Ab24733) followed by 1:500 dilution of secondary antibodies conjugated with 555, 488, or 647 fluorochromes (Invitrogen, A131570, A312572, A21202, A21206, A21141, A31571, A31573, A21240 or A21447). Immunostained and unstained single cell samples at a density of 20 000 cells/μl in in 0.1M TBS wash buffer with 1% fish gelatin were imaged using Imagestream Mk II Imaging Flow Cytometer (Luminex). Number of cells in-focus per each biologically independent experiment or timepoint has been reported in the Figure legends.

For flow cytometry, cells were detached using Trypsin/EDTA (Cell Applications), resuspended in PBS supplemented with 10% HSA (Octapharma), blocked with FcR blocking reagent (Miltenyi Biotec, 130-059-901), and incubated with pre-conjugated CD133/1 (AC133) PE, human antibody (Miltenyi Biotec, 130-113-108) and CD34 (AC136) FITC, human antibody (Miltenyi Biotec, 130-113-178) for 30 min at 4°C. The cells were washed with PBS-10% HAS, stained with 1μg/mL DAPI (4',6-Diamidino-2-Phenylindole, Dilactate) (Biolegend, 422801) for viability. For mitochondria membrane potential or ROS analysis, cells were incubated either with 100nM MitoTracker® Orange CM-H2TMRos dye (Invitrogen, Cat. no. M7511) or 5 μM carboxy-H2DCFDA (Invitrogen, Cat. no. C400) and 1:1000 propidium iodide (Invitrogen, P3566) live stain before analysis using a BD FACSDiva 9 Flow Cytometer (BD Biosciences).

Instrument

Amnis® ImageStream®x Mark II Imaging Flow Cytometer (Luminex), and BD FACSDiva 9.0 Flow Cytometer (BD Biosciences)

Software

Data analysis was done using either IDEAS software version 6.2, FlowJo version 10.10.0. or FCS Express version 6 (De Novo Software)

Cell population abundance

For imaging flow cytometry analysis, number of cells in-focus per each biologically independent experiment or timepoint has been reported in the Figure legends. Flow analysis for CD133, MitoTracker® Orange CM-H2TMRos, or carboxy-H2DCFDA was performed by collecting a minimum of 100,000 events per sample.

Gating strategy

mScarlet- (i.e. BAI1 WT) and mScarlet+ (i.e. BAI1 KO) hNSC were separated and enriched using a BD Fusion Cell Sorter (BD Biosciences). Gating for the FACS were set using cells negative for mScarlet. The gating parameters were highly stringent to avoid any heterozygous cell populations (low expression of BAI1) within the sorted cell pools.

Gating for the Imagestream flow cytometry analyses were set using unstained negative control cells, 2. antibody only controls, single labeled cells, and antibody compensation beads (Life technologies, A10513). Schematic examples of Imagestream imaging flow cytometer gating for C1q internalization, BAI1-C1q intracellular co-localization and intracellular C1q spot counts using IDEAS software are shown in Supplementary Fig. 3b,c.

For flow analysis for CD133, MitoTracker® Orange CM-H2TMRos, or carboxy-H2DCFDA gating were set using unstained negative control cells, 2. antibody only controls, single labeled cells, and antibody compensation beads.

☒ Tick this box to confirm that a figure exemplifying the gating strategy is provided in the Supplementary Information.
